# Interaction between Aβ and tau on reversion and conversion in mild cognitive impairment patients: After 2-year follow-up

**DOI:** 10.1016/j.heliyon.2024.e26839

**Published:** 2024-02-21

**Authors:** Jinzhi Tang, Qiuping Chen, Zhenfa Fu, Yuqun Liang, Guohua Xu, Huan Zhou, Bingjie He, Alzheimer's Disease Neuroimaging Initiative

**Affiliations:** a Neurological Function Examination Room, The First Affiliated Hospital of Jinan University, Guangzhou, PR China; b Department of Rehabilitation, Guangzhou Panyu Health Management Center (Guangzhou Panyu Rehabilitation Hospital), Guangzhou, PR China

**Keywords:** Mild cognitive impairment, Amyloid-β, Tau, Reversion, Conversion

## Abstract

**Background:**

The role of amyloid-β (Aβ) and tau in reversion and conversion in patients with mild cognitive impairment (MCI) remains unclear. This study aimed to investigate the influence of cerebrospinal fluid (CSF) Aβ and tau on reversion and conversion and the temporal sequence of their pathogenicity in MCI patients.

**Methods:**

179 MCI patients were recruited from the Alzheimer's Disease Neuroimaging Initiative database and classified into two groups based on cognitive changes after follow-up: reversal group (MCI to cognitively normal) and conversion group (MCI to Alzheimer's disease). CSF biomarkers and cognitive function were measured at baseline and 2-year follow-up. Partial correlation was used to analyze the association between CSF biomarkers and cognitive function, and multivariable logistic regression to identify independent risk factors for cognitive changes at baseline and 2-year follow-up. Receiver operating characteristic (ROC) curves were utilized to evaluate the predictive ability of these risk factors for cognitive changes.

**Results:**

The differences in cognitive function and CSF biomarkers between the two groups remained consistent with baseline after 2-year follow-up. After controlling for confounding variables, there was still a correlation between CSF biomarkers and cognitive function at baseline and 2-year follow-up. Multivariable regression analysis found that at baseline, only Aβ level was independently associated with cognitive changes, while Aβ and tau were both predictive factors after 2-year follow-up. ROC curve analysis revealed that the combination of Aβ and tau [area under the curve (AUC) 0.91, sensitivity 84%, specificity 86%] in predicting cognitive changes after 2-year follow-up had better efficacy than baseline Aβ alone (AUC 0.81).

**Conclusion:**

Aβ may precede Tau in causing cognitive changes, and the interaction between the two mediates cognitive changes in patients. This study provides new clinical evidence to support the view that Aβ pathology precedes tau pathology, which together contribute to the changes in cognitive function.

## 1 Introduction

Mild cognitive impairment (MCI) is considered an intermediate stage between normal cognition and dementia, with an incidence rate of about 5%–30% [5,18]. With the global aging population, this proportion is expected to increase further, leading to a rise in the number of individuals diagnosed with clinical Alzheimer's disease (AD). This, in turn, will significantly impact healthcare systems and families worldwide [19]. To alleviate this burden, researchers have conducted numerous studies to explore interventions aimed at preventing the progression of MCI. However, these studies have demonstrated limited effectiveness in improving cognitive function through interventions such as exercise, cognitive training, sleep-improving medication, acetylcholinesterase inhibitors, and excitatory amino acid receptor antagonists [2,26]. Consequently, clinical trials aimed at treating AD have, thus far, yielded disappointing results [22].

Previous studies have provided evidence suggesting a relationship between CSF biomarkers, such as amyloid-β (Aβ) and total tau, and the risk of developing MCI or AD from a state of normal cognition [25]. Additionally, abnormal changes in the levels of these biomarkers have been observed as the disease progresses [14]. However, it remains unclear whether these changes are independently associated with cognitive function changes, and few studies have specifically investigated the transition from MCI to cognitively normal (CN). This knowledge gap might be attributed to a lack of sufficient longitudinal follow-up data in most studies. However, the use of the Alzheimer's Disease Neuroimaging Initiative (ADNI) database allows for feasible analyses in this regard. The ADNI database includes participants who were initially diagnosed with MCI and were subsequently followed up for several years, with CSF biomarker levels and cognitive function scores recorded at multiple time points.

The primary aim of this investigation is to determine which CSF biomarkers, obtained from individuals diagnosed with MCI at baseline, accurately predict changes in cognitive function during subsequent progression to either AD or CN, and whether these biomarkers induce changes in a sequential manner. To evaluate the diagnostic accuracy of the measurements for predicting cognitive changes at different follow-up times (i.e., baseline and 2 years after baseline), a receiver operating characteristic (ROC) analysis will be conducted. This analysis will help evaluate the ability of the measured biomarkers to accurately predict changes in cognitive function over time.

## 2 Methods

### 2.1 ADNI study

The data utilized in this investigation were obtained from the ADNI database (adni.loni.usc.edu), which was established as a public-private partnership in 2003, under the guidance of Principal Investigator Michael W. Weiner, MD. Currently, the database primarily consists of CSF biomarkers, magnetic resonance, and other biomarker data derived from individuals with CN, MCI, and AD. Additional information can be accessed on the website www.adni-info.org. Each ADNI site received approval from the Institutional Review Board (IRB). Written informed consent was provided by all participants or authorized representatives.

### 2.2 Subjects

For this study, a total of 179 participants with MCI were recruited. Out of these, 132 individuals progressed to a diagnosis of AD, while 47 individuals showed cognitive improvement and transitioned to a CN state. The diagnostic criteria were described in detail in the manual of the ADNI program [24]. The diagnosis of CN required older individuals with normal cognitive function that matched their age, gender, and education level. The diagnostic criteria for MCI were as follows: Mini-Mental State Examination (MMSE) scores ranging from 24 to 30, the presence of memory complaints, objective memory loss as measured by education-adjusted scores on Wechsler Memory Scale Logical Memory II, a Clinical Dementia Rating of 0.5, the absence of significant levels of impairment in other cognitive domains, essentially preserved activities of daily living, and no evidence of dementia. A definitive diagnosis of AD required a combination of dementia syndrome and the diagnostic criteria established by the National Institute of Neurological and Communicative Disorders and Stroke/Alzheimer's Disease and Related Disorders Association (NINCDS/ADRDA) [15].

### 2.3 Neuropsychological assessment

In this study, we employed a battery of standardized assessments to comprehensively evaluate cognitive function and daily living ability among our study participants. Specifically, we used the Mini-Mental State Examination (MMSE) to assess overall cognitive function, the 13-item version of the Alzheimer's Disease Assessment Scale-Cognitive subscale (ADAS-cog13) to measure cognitive impairment degree, the Rey Auditory Verbal Learning Test (RAVLT) immediate recall to assess verbal memory, and the Functional Activities Questionnaire (FAQ) to evaluate daily living ability.

### 2.4 Measurement of CSF Aβ42, tau, and P-tau

The concentrations of CSF Aβ, tau, and P-tau were quantified using the INNOBIA AlzBio3 kit (Fujirebio, Ghent, Belgium) and the multiplex xMAP Luminex platform (Luminex Corp, Austin, TX, USA) at the ADNI biomarker core located at the University of Pennsylvania, as previously described [21].

### 2.5 Apolipoprotein E genotyping

Comprehensive information about the Apolipoprotein E (ApoE) genotypes in this study can be accessed at adni.loni.usc.edu. In this study, individuals who carried at least one ε4 allele were classified as ApoE ε4 positive.

### 2.6 Measurement of brain tissue volume

The ADNI neuroimaging standardized procedure has been extensively described elsewhere [11]. T1-weighted sagittal 3D magnetization-prepared rapid gradient-echo sequences were used to acquire ADNI MRI data on 1.5 or 3 T MRI scanners. Cortical reconstruction and volumetric segmentation were performed using FreeSurfer version 5.1 image analysis suite, which has been previously described in other reports [11,13]. This study focused on the evaluation of ventricular, hippocampal, entorhinal cortex, fusiform, and medial temporal lobe volumes. Further information regarding ADNI imaging protocols is available at http://adni.loni.usc.edu/methods/documents/mri-protocols/.

### 2.7 Statistical analysis

In this study, data that adhered to a normal distribution were presented as mean ± standard deviation (SD), while the results of skewed distribution analyses were conveyed as median (M) and interquartile range (IQR). Ratios were articulated as percentages. The Chi-square test was implemented to appraise the differences in gender, marital status, and ApoE positivity between the reversal and conversion group. Mann-Whitney *U* test was utilized to evaluate the differences in education, cognitive function scores, and CSF biomarkers among two groups. Partial correlation analysis was conducted to scrutinize the association between CSF biomarkers and cognitive function scores at baseline and at the 2-year follow-up, controlling for age and ApoE. The correlation coefficient was represented by r_s_. Multivariable logistic regression was employed to identify independent risk factors for cognitive changes at baseline and the 2-year follow-up. Statistical analyses were performed employing SPSS software (version 26.0; IBM SPSS). ROC curves were generated using Medcalc to compare the predictive accuracy of individual CSF biomarkers and diverse combinations of biomarkers for cognitive changes. The significance level for all calculated tests was set at *P* < 0.05.

## 3 Results

### 3.1 Baseline features

A total of 179 participants were included, comprising 47 individuals who transitioned from MCI to CN (reversal group) and 132 individuals who progressed from MCI to AD (conversion group). Compared to the reversal group, patients in the conversion group did not show any differences in gender, educational level, or marital status, except for being older in age. However, significant differences were observed in ApoE, cognitive function scores, brain tissue volume, and CSF biomarker levels (Table 1). Interestingly, the median time for individuals in both groups to progress from MCI to AD or recover to CN was 2 years.

**Table 1:** Demographics and clinical characteristics of MCI patients progressing to CN or AD at baseline.

Characteristics	Reversal group (n = 47)	Conversion group (n = 132)	*P* value
Age, years	68.2 ± 7.9	73.5 ± 7.1	＜0.001
Gender, n (% Female)	24 (51.1)	50 (37.9)	0.117
Education, years	17 (15–18)	16 (14–18)	0.081
Marital status, n (% Married)	36 (76.6)	115 (87.1)	0.092
ApoE ε4 carriers, n (%)	21 (44.7)	83 (62.9)	0.031
Cognitive conversion time, years	2 (1–3)	2 (1–3)	0.577
MMSE	29 (29–30)	27 (25–28)	＜0.001
RAVLT-immediate recall	46 (37–51)	28 (24–34)	＜0.001
FAQ	0 (0–1)	4 (2–8)	＜0.001
ADAS 13	9 (7–14)	20 (16–24)	＜0.001
Ventricles	23922.0 (18441.0–33979.5)	42129.0 (29073.0–57111.0)	＜0.001
Hippocampus	7655.0 (7291.5–7997.0)	6021.0 (5524.5–6959.5)	＜0.001
Entorhinal	3978.0 (3454.5–4291.5)	3105.0 (2709.0–3651.0)	＜0.001
Fusiform	18447.0 (17206.0–20214.0)	16815.0 (14633.0–18354.0)	＜0.001
middle temporal	20622.0 (18991.5–23303.5)	18704.0 (16415.0–20852.0)	＜0.001
CSF Aβ (pg/ml)	1271.0 (871.9–1700.0)	634.9 (534.0–790.6)	＜0.001
CSF tau (pg/ml)	230.5 (171.5–257.0)	327.5 (251.2–426.1)	＜0.001
CSF P-tau (pg/ml)	20.0 (14.9–24.0)	32.6 (23.9–43.7)	＜0.001

CN, cognitively normal; MCI, mild cognitive impairment; AD, Alzheimer's disease; ApoE, apolipoprotein E; MMSE, Mini-Mental state examination; RAVLT, Rey auditory verbal learning test; FAQ, functional activities questionnaire; ADAS13, Alzheimer's Disease Assessment Scale-Cognitive Subscale 13; CSF, cerebrospinal fluid; Aβ, amyloid-β.

### 3.2 Cognitive function and biomarkers at the 2-year follow-up

After screening patients with 2-year follow-up for CSF biomarkers, there were 14 cases in the reversal group and 75 cases in the conversion group. The follow-up data after two years showed that compared with the reversal group, the conversion group had lower MMSE and RAVLT-immediate recall scores, and higher FAQ and ADAS13 scores, indicating that the conversion group had more severe cognitive impairment, language memory and daily life ability impairment (all *P* < 0.01, Table 2). CSF testing showed that the levels of Aβ and tau in the conversion group were lower than those in the reversal group, while P-tau was higher than that in the reversal group (all *P* < 0.05, Table 2).

**Table 2:** Cognitive function scores and CSF biomarkers of MCI patients progressing to CN or AD at two-year follow-up.

	Reversal group (n = 14)	Conversion group (n = 75)	*P* Value
**MMSE**	29 (28–30)	25 (22–26)	＜0.001
**RAVLT-immediate recall**	46 (39–53)	25 (20–32)	＜0.001
**FAQ**	0.6 (0–1)	11 (5–17)	0.003
**ADAS13**	7 (6–10)	26 (19–32)	＜0.001
**CSF Aβ(pg/ml)**	1236.5 (706.0–1700.0)	605.0 (491.9–744.7)	＜0.001
**CSF tau (pg/ml)**	243.0 (199.7–259.6)	343.2 (258.8–480.8)	0.005
**CSF P-tau (pg/ml)**	21.0 (17.7–26.6)	33.5 (24.5–47.0)	0.005

CN, cognitively normal; MCI, mild cognitive impairment; AD, Alzheimer's disease; MMSE, Mini-Mental state examination; RAVLT, Rey auditory verbal learning test; FAQ, functional activities questionnaire; ADAS13, Alzheimer's Disease Assessment Scale-Cognitive Subscale 13; CSF, cerebrospinal fluid; Aβ, amyloid-β.

In addition, we compared the cognitive function and CSF biomarker levels at different time points within the two groups. In the conversion group, there was a significant decline in MMSE (*P* = 0.011) and RAVLT-immediate recall scores (*P* = 0.004), and a significant increase in ADAS13 scores (*P* = 0.001) compared to baseline after a 2-year follow-up, while there was no significant change in FAQ scores (*P* = 0.193). In the reversal group, there was no significant change in MMSE, RAVLT-immediate recall, ADAS13, and FAQ compared to baseline after a 2-year follow-up (all *P* > 0.05). In the conversion group, there may be a decreasing trend in Aβ levels compared to baseline after a 2-year follow-up (*P* = 0.053), while there was no significant change in tau and P-tau (all *P* > 0.05). Similarly, in the reversal group, there was no statistically significant difference in changes of CSF biomarkers (all *P* > 0.05).

### 3.3 Correlation analysis between biomarkers and cognitive function

To reduce the influence of age and APOE on cognitive function, we conducted a partial correlation analysis to control for these two factors. Table 3 showed that at baseline, Aβ was weakly positively correlated with MMSE (r_s_ = 0.29, *P* ＜0.001) and RAVLT-immediate recall (r_s_ = 0.37, *P* ＜0.001), and weakly negatively correlated with FAQ (r_s_ = −0.20, *P* = 0.006) and ADAS13 (r_s_ = −0.44, *P* ＜0.001), of which similar results were observed after a 2-year follow-up. At baseline and after a 2-year follow-up, tau and P-tau had a weak negative correlation with MMSE and RAVLT-immediate recall, and positive correlation with ADAS13 (all *P* < 0.05, see Table 3).

**Table 3:** Partial correlation analysis between biomarkers and cognitive function

**Characteristics**	Baseline			Two-year follow-up		
	CSF Aβ r_s_ (*P* value)	CSF tau r_s_ (*P* value)	CSF P-tau r_s_ (*P* value)	CSF Aβ r_s_ (*P* value)	CSF tau r_s_ (*P* value)	CSF P-tau r_s_ (*P* value)
**MMSE**	0.29 (＜0.001)	-0.23 (0.002)	-0.24 (0.001)	0.25 (0.018)	-0.26 (0.014)	-0.21 (0.046)
**RAVLT-immediate recall**	0.37 (＜0.001)	-0.26 (＜0.001)	-0.27 (＜0.001)	0.23 (0.037)	-0.31 (0.003)	-0.30 (0.004)
**FAQ**	-0.20 (0.006)	0.23 (0.001)	0.13 (0.078)	-0.24 (0.029)	0.18 (0.093)	0.13 (0.225)
**ADAS13**	-0.44 (＜0.001)	0.30 (＜0.001)	0.31 (＜0.001)	-0.26 (0.016)	0.36 (＜0.001)	0.30 (＜0.001)

MMSE, Mini-Mental state examination; RAVLT, Rey auditory verbal learning test; FAQ, functional activities questionnaire; ADAS13, Alzheimer's Disease Assessment Scale-Cognitive Subscale 13; CSF, cerebrospinal fluid; Aβ, amyloid-β.

### 3.4 Prediction of cognitive changes

Based on binary logistic regression, the variables that were independently associated with cognitive changes were only CSF Aβ [(odds ratio (OR) = 0.99, 95% confidence interval (CI) = 0.98–0.99, *P* < 0.0001)] and ApoE ε4 (OR = 0.17, 95% CI = 0.04–0.76, *P* = 0.020) at baseline, while Aβ (OR = 0.99, 95% CI = (0.98–0.99), *P* = 0.015), tau (OR = 1.07, 95% CI = (1.00–1.14), *P* = 0.020) and age (OR = 1.19, 95% CI = 1.00–1.42, *P* = 0.045) were predictive factors after 2-year follow-up (Table 4).

**Table 4:** Multivariable logistic regression of cognitive changes at baseline and 2-year follow-up.

Characteristics	Baseline		Two-year follow-up	
	Adjusted OR (95 % CI)	*P* value	Adjusted OR (95 % CI)	*P* value
Age	1.06 (0.99–1.13)	0.060	1.19 (1.00–1.42)	0.045
ApoE ε4	0.17 (0.04–0.76)	0.020	0.05 (0.01–2.01)	0.112
CSF Aβ	0.99 (0.98–0.99)	＜0.001	0.99 (0.98–0.99)	0.015
CSF tau	1.02 (0.99–1.05)	0.204	1.07 (1.00–1.14)	0.036
CSF P-tau	0.94 (0.74–1.19)	0.609	0.60 (0.35–1.04)	0.071

MCI, mild cognitive impairment; AD, Alzheimer's disease; ApoE, apolipoprotein E; CSF, cerebrospinal fluid; OR, odds ratio; CI, conﬁdence interval; CSF, cerebrospinal fluid; Aβ, amyloid-β.

**Fig. 1:**
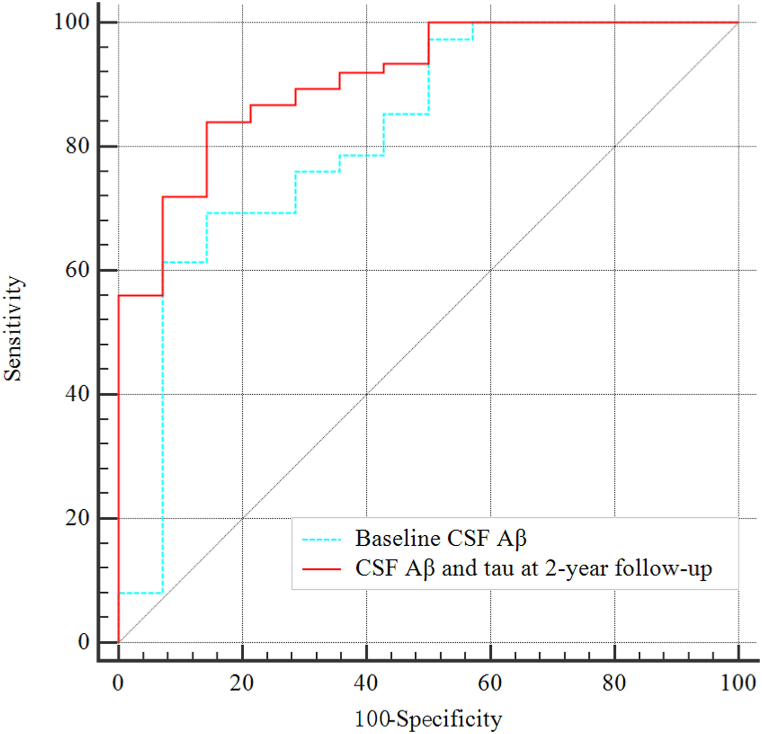
Receiver operating characteristic curves with the quantitative variables independently associated with cognitive changes. CSF, cerebrospinal fluid; Aβ, amyloid-β.

Fig. 1 shows the ROC curve for Aβ at baseline or the combing of Aβ and tau at 2-year follow-up to identify cognitive changes. The optimal threshold of Aβ at baseline for cognitive changes were 775.6 pg/ml [area under the curve (AUC) = 0.81, 95% CI = 0.72–0.89, *P* < 0.0001, sensitivity 69%, specificity 85%]. Moreover, combing of Aβ and tau at 2-year follow-up reached AUC of 0.91 (95% CI = 0.83–0.96, *P* < 0.0001, sensitivity 84%, specificity 86%) with the best cut-off values of 524.2 pg/ml and 332.3 pg/ml, respectively, and the combination had better efficacy than baseline Aβ alone (*P* = 0.041).

## 4 Discussion

In this study, we observed differences in cognitive function and CSF biomarkers between the reversal and conversion group at baseline and 2-year follow-up. Furthermore, we have identified a correlation between CSF biomarkers and cognitive function at both the baseline evaluation and the 2-year follow-up. Importantly, our findings indicate that at the initial assessment, only the level of Aβ was independently associated with cognitive changes. However, following the 2-year follow-up, both Aβ and tau levels demonstrated independent associations with cognitive changes. Remarkably, ROC curve analysis also showed that the combination of Aβ and tau was more accurate in predicting cognitive changes after 2-year follow-up than utilizing baseline Aβ levels alone.

MCI serves as an intermediate stage between normal cognition and dementia, with the potential to progress to dementia or revert back to normal cognition. Clinically, the conversion rate of MCI to dementia is approximately 14% and tends to increase with longer follow-up periods [23]. Conversely, meta-analysis suggests that the reversion rate of MCI to normal cognition is around 25% after a follow-up period exceeding 2 years [4]. The presence of Aβ and tau proteins plays a crucial role in the development and progression of MCI and AD. Extensive basic research, involving in vivo and in vitro experiments, has established that inhibiting abnormal Aβ deposition and excessive tau phosphorylation can potentially reverse cognitive deficits in AD models [7]. Through a 1 to 2-year follow-up of 80 MCI subjects, 3 individuals reverted to normal cognition, and it was found that compared to stable MCI patients, the reversal group had higher levels of CSF Aβ, while levels of tau and P-tau were lower [3]. Additionally, another clinical study observed that the reversal group had the highest levels of CSF Aβ and the lowest levels of tau and P-tau, in contrast to the stable MCI and MCI conversion groups [1]. Consistent with prior research [1,3], our study also found that at baseline, the reversal group had higher levels of CSF Aβ and lower levels of tau and P-tau compared to the MCI conversion group. Remarkably, these differences in CSF biomarkers persisted in both groups during the 2-year follow-up period. Furthermore, we discovered that the frequency of ApoE ε4 in the reversal group was lower than that in the conversion group, aligning with previous study observations [1].

Given the observed differences in CSF biomarkers between the conversion and reversal groups, it is worthwhile to investigate the association between these differences and cognitive function. Evidently, cognitive impairment in the reversal group was lower compared to the conversion group, as indicated by various cognitive function scores. Furthermore, our study revealed a positive correlation between Aβ levels and good cognitive function, while tau and P-tau levels exhibited a negative correlation with good cognitive function. This can be attributed to the mechanism wherein Aβ and tau contribute to neuronal loss and synaptic damage, subsequently leading to a decline in cognitive function in patients [10,16]. As a result, it is reasonable to infer that Aβ levels are positively correlated with cognitive function, and the reversal group, having better cognitive function, exhibits higher Aβ levels, which is supported by our data. Similarly, it can be inferred that the reversal group should also demonstrate higher levels of tau and P-tau, and our data supports this inference.

Numerous factors influence changes in cognitive function. Engaging in specific lifestyles, such as field work or gardening, has shown potential to reverse MCI [17]. Conversely, factors like stress, sleep disturbances, and nutritional deficiencies can promote the conversion of MCI to AD [20]. At the molecular level, our study found that only Aβ could predict the conversion of MCI to AD at baseline, whereas both Aβ and Tau could predict this conversion after a 2-year follow-up. This provides robust evidence for the time-ordered correlation of Aβ and Tau with MCI progression, supporting previous observations of differential secretion levels of these biomarkers [8,9]. Furthermore, we discovered that combining Aβ and Tau yielded higher accuracy in predicting the conversion and reversal of MCI compared to using Aβ alone. This can be attributed to the cascade changes of biomarkers from Aβ to Tau, following a specific order during the conversion of MCI to AD, which subsequently leads to neurodegeneration [12]. When CSF levels of Aβ are high and Tau levels are low, MCI patients are more likely to experience a reversal to normal cognitive function. Conversely, when Aβ levels are low and Tau levels are high, they are more likely to convert to AD. The potential mechanisms underlying the ability of physical activity, dietary changes, cognitive training, and other interventions to reverse MCI may involve altering the secretion levels of CSF biomarkers [6].

Our study had several limitations that should be acknowledged. Firstly, due to the invasive nature of CSF biomarker testing, some patients did not undergo this procedure during the 2-year follow-up period. This could potentially introduce bias to the study results. It is important to note that patient consent and individual preferences may have influenced the decision to undergo CSF biomarker testing, particularly among those experiencing MCI reversal. Secondly, our study did not include MCI stabilizers as a comparison group. Therefore, we were unable to assess the differences in CSF biomarker levels between MCI stabilizers, reversers, and converters. However, this limitation does not impact the overall conclusions drawn from our study. Lastly, we did not collect data on factors that could influence MCI conversion and reversal, such as patients' neuroimaging data and daily habits. These factors could provide valuable insights into the mechanisms underlying MCI progression and reversal.

## 5 Conclusion

Our findings suggest that Aβ may play a role before Tau in driving cognitive changes in MCI patients. Moreover, the combination of Aβ and Tau demonstrates superior predictive accuracy for MCI conversion and reversal compared to using Aβ alone. In order to validate our findings and provide more robust evidence, larger longitudinal follow-up studies should be conducted in the future. This will allow for a more comprehensive analysis of the relationship between Aβ, Tau, and cognitive changes in MCI patients.

## Statements

### Statement of ethics

All procedures performed in the study involving human participants were in accordance with the ethical standards of the institutional and/or national research committee and with the 1964 Helsinki Declaration and its later amendments or comparable ethical standards. Written informed consents were obtained from all participants or authorized representatives included in the study.

## Funding

This work was supported by the Guangzhou Health and Technology Project (20231A041009) and the Key Medical and Health Project of Panyu District Science and Technology Plan (2022-Z04-106).

## Data availability statement

Researchers interested in accessing the ADNI data can do so through the LONI Imaging & Data Archive. To apply for access, they can visit the ADNI website at http://adni.loni.usc.edu/data-samples/access-data/.

## CRediT authorship contribution statement

**Jinzhi Tang:** Writing – original draft, Formal analysis, Conceptualization. **Qiuping Chen:** Writing – original draft, Software, Data curation, Conceptualization. **Zhenfa Fu:** Supervision. **Yuqun Liang:** Methodology. **Guohua Xu:** Supervision. **Huan Zhou:** Writing – review & editing, Supervision, Funding acquisition, Conceptualization. **Bingjie He:** Writing – review & editing, Resources, Funding acquisition, Conceptualization.

## Declaration of competing interest

The authors declare that they have no known competing financial interests or personal relationships that could have appeared to influence the work reported in this paper.
